# Locating neural transfer effects of *n*-back training on the central executive: a longitudinal fMRI study

**DOI:** 10.1038/s41598-020-62067-y

**Published:** 2020-03-23

**Authors:** Anna Miró-Padilla, Elisenda Bueichekú, César Ávila

**Affiliations:** Neuropsychology and Functional Neuroimaging Group, Department of Basic Psychology, Clinical Psychology and Psychobiology, Universitat Jaume I. Avda. Sos Baynat, s/n, 12071 Castelló, Spain

**Keywords:** Human behaviour, Cognitive neuroscience, Cortex, Working memory, Cortex

## Abstract

The large number of behavioral studies testing whether working memory training improves performance on an untrained task have yielded inconclusive results. Moreover, some studies have investigated the possible neural changes during the performance of untrained tasks after training. Here, we studied the transfer from *n*-back training to the Paced Auditory Serial Addition Test (PASAT), two different tasks that use the central executive system to maintain verbal stimuli. Participants completed fMRI sessions at baseline, immediately after one week of training, and at the five-week follow-up. Although behavioral transfer effects were not obtained, training was associated with decreased activation in the anterior dorsolateral prefrontal cortex (DLPFC; BA 9/46) while performing the PASAT that remained stable five weeks later. Consistent with our hypothesis, the changes in the anterior DLFPC largely overlapped with the *n*-back task fMRI activations. In conclusion, working memory training improves efficiency in brain areas involved in the trained task that may affect untrained tasks, specifically in brain areas responsible for the same cognitive processes.

## Introduction

The changes that cognitive training produce in the brain and in behavior have been extensively researched. Particularly, in recent years, working memory training has been studied by a large number of researchers, but with no agreement about its behavioral and neural effects on untrained tasks. Although it seems clear that working memory training leads to improvements in the trained tasks due to practice (see e.g.^1,2^ for reviews), the controversy arises in relation to the possible transfer effects to untrained tasks. This transfer effect means that practicing one cognitive process would facilitate the performance of similar tasks using the same process. In this field, the distinction between near and far transfer remains unclear. Some authors distinguish near and far transfer depending on the structural similarity between the trained and untrained working memory tasks^3–5^: near transfer is produced between structurally similar or identical tasks, and far transfer occurs between dissimilar working memory tasks. However, other researchers define near and far transfer applied to different cognitive domains: near transfer when moving within the same cognitive domain and far transfer when moving across other cognitive domains^2,6,7^. We will adopt the first definition throughout this study.

As mentioned above, there is no agreement because, whereas some studies have reported near and far transfer effects^8–10^, others have only found near transfer effects^11–14^, and still others did not find any kind of transfer^15–18^. At least five meta-analyses^5,7,19–21^ have been conducted to clarify the possible transfer effects in the working memory domain, but the overall results have been inconclusive. The most recent review by Soveri *et al*.^7^ reported a moderate effect of task-specific transfer, but very small near and far transfer effects (the same or different cognitive domains). Furthermore, this last study did not find a moderating role in the results of the kind of control group, the training type, the number of sessions, or the hours of training. Therefore, other non-behavioral approaches should be used to investigate transfer effects after working memory training. A further interesting point is the existence of cross-modal effects on transfer^22^. A very elegant experiment demonstrated the existence of this transfer, but only when training in the dominant sensory modality (i.e. visual for visuospatial task and auditory for a temporal task) benefits the performance in a non-dominant sensory modality^23^.

At the neural level, working memory is mainly supported by a distributed frontoparietal set of cortical regions^24^. The intraparietal and superior parietal cortex and the posterior parts of the superior frontal gyrus are more involved in visuospatial working memory tasks, whereas the more anterior parts of the prefrontal cortex act as the central executive and are crucial for storing non-spatial information and exerting top-down control over posterior regions. The top-down control exerted by the anterior parts of the prefrontal cortex has been observed across a variety of cognitive tasks, independently of the complexity or cognitive demands^25,26^.

Apart from the large number of working memory behavioral studies, there is little research examining the cerebral changes produced by working memory training. Recent scientific reviews on this topic describe the brain changes after different kinds of training^24,27,28^. Again, disagreement is found because both increases and decreases in task-related BOLD activity have been described after training in working memory brain related areas, especially in frontoparietal areas. However, in the specific case of *n*-back training, most investigations (see for instance:^10,29–32^) showed decreased activation after training in the areas related to the *n*-back task, which are the superior middle frontal cortex (BA 6, right hemisphere), posterior parietal regions (BA 40), and dorsolateral prefrontal cortex (DLPFC; BA 9, 46). Thus, the effect of *n*-back training on efficiency on the same task leads to improved performance and a reduction in the activation in frontoparietal areas. There is evidence showing that this effect is cross-modal. In a recent study, visual or tactile training on texture discrimination produces similar improvement, and using the same brain areas, on the visual discrimination test^22^. These cross-modal effects have been shown to be directional in the sense that training on a visuospatial *n*-back task improves the performance on auditory *n*-back^8^, but not in the opposite direction (auditory *n*-back training did not improve visual *n*-back^33^).

Research focused on the study of transfer effects in the brain is still not very well developed. In a very recent meta-analysis, the authors claimed that the low number of transfer studies made it difficult to draw general valid conclusions^28^. In a study that compared HIV patients to healthy controls, participants carried out an adaptive training on an *n*-back task and completed three fMRI sessions, performing 1- and 2-back tasks^29^. The results showed improvements in performance and decreased activation in frontoparietal areas in both groups at the follow-up sessions. Importantly, the decreased activation observed in the DLPFC while performing the 2-back task correlated with behavioral improvements on a short-term memory task (i.e. Digit Span task, performed outside the scanner) in the HIV group, whereas both groups had decreases in activation in the middle frontal cortex during the 1-back task that correlated with short-term behavioral improvements. Therefore, the decreased activation in frontal areas on the trained task was related to near transfer. However, another very recent study did not replicate these results when transfer effects to a fluid intelligence test were studied^30^. In Dahlin *et al*.^31^, for five weeks, participants received computer-based updating training on a letter recall task requiring the discrete updating of four letters. *N*-back and Stroop tests were included as transfer tasks. Analyses of post-training effects in fMRI while performing the task revealed enhanced activation in temporal structures, occipital areas, and in the left basal ganglia (striatum), but less activation in frontal and parietal regions. The near transfer effects to the *n*-back task were seen in the left striatum cortex, and no significant transfer effects to the Stroop task were found. The authors confirmed the hypothesis that transfer effects occur when the training task and the transfer task involve the same brain regions and processing components. A more recent study found increases in the occipital cortex and the striatum while performing a near transfer task, even on trained and untrained tasks requiring the updating of working memory^32^. Importantly, the neural transfer was observed in the dual-task condition, but not in the single task conditions because it was specific to the process of updating two different stimuli.

Adopting the structural definition of near and far transfer, that is, when transfer is produced between structurally similar or dissimilar working memory tasks, the aim of this study was to investigate neural far transfer processes between two dissimilar working memory tasks: the *n*-back and the Paced Auditory Addition Test (PASAT). We chose the auditory version instead of the visual version of the PASAT because it is more popular and activations in working memory brain areas were similar^34^. Considering that the visual domain is dominant in *n*-back training^8^, we may observe a pure cross-modal effect of cognitive processes of working memory to the auditory PASAT, whereas the use of the visual version of the PASAT would allow us to also attribute the transfer to the visual function. Both the *n*-back and PASAT tasks are common experimental working memory paradigms that continuously require keeping information available for subsequent complex processing^35–37^, and both require the participation of the DLPFC^37–39^. They also have the premotor cortex, the dorsal anterior cingulate cortex, and the posterior parietal cortex^34,37–39^ in common during the execution of the task. Both tasks require the person to maintain and update past information, However, it must be highlighted that these two tasks involve different posterior cognitive processes: the *n*-back task requires the subject to maintain letters, whereas the PASAT requires him/her to maintain a number, resist the interference of a generated number, and add it to the actual number.

Through a longitudinal fMRI study, the present study investigated the possible transfer effect from *n*-back training to PASAT at the behavioral and brain levels. In addition, for the purposes of this investigation, the possible reliability of the changes was studied in a follow-up session in order to obtain valuable information about the effect of training over time. Our hypotheses were that we would observe a transfer effect consisting of: (1) a behavioral improvement on the PASAT task; and (2) a neural effect involving reduced activation in the DLPFC, which would mediate top-down control on both tasks. The DLPFC is the main brain area involved in non-spatial working memory processing^24^, and it has been involved in transfer effects in previous research^28,29^. Posterior brain areas were not expected to be sensitive to transfer effects because they have different roles in the two tasks (maintain letters in memory vs add/manipulate numbers).

## Results

### Accuracy results

#### Accuracy results during fMRI sessions

**Figure 1:**
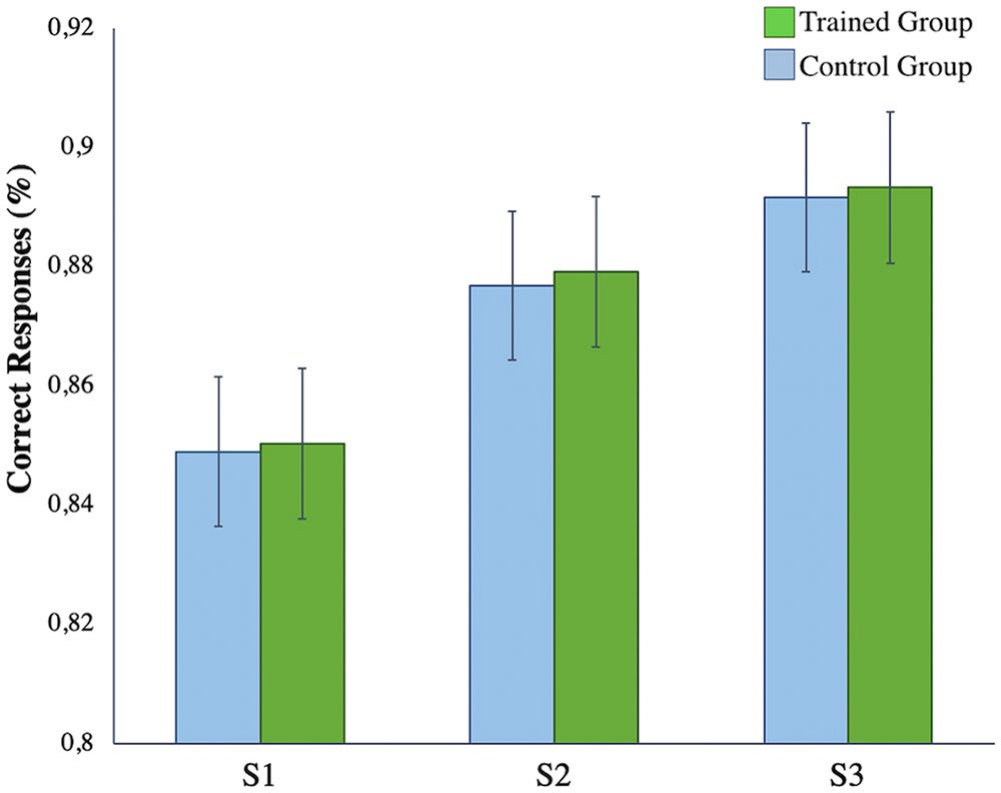
Results of the PASAT behavioral analysis. Differences were found between sessions, but not between groups. Correct-responses percentage. Pre-training session is Session 1 (S1), post-training session is Session 2 (S2) and follow-up session is Session 3 (S3). Green bars match to trained group data and blue bars to control group data. Error bars represent standard error. N = 52 (Trained group = 25; Control group = 27).

Mean performance on the pre-training was close to 85% for both groups (Fig. 1). An analysis of variance (ANOVA) with Session as within-subject factor (3 levels: S1, S2 and S3) and Group as between-subject factor (2 levels: Trained group and Control group) was performed with the accuracy data from the PASAT test. A main effect was found for Session (*F*_(2,48)_ = 7.72, *p-value* = 0.001), with better accuracy after training (S2) and in the follow-up (S3). Importantly, the main effect of Group and the Group × Session interaction were non-significant, showing that the trained group did not perform the task significantly better than the control group after *n*-back training. In addition, the ANOVA conducted to investigate the effects of training on the *n*-back task yielded significant differences between groups and sessions, indicating improvements in terms of accuracy and reaction times (RTs) after training that remained stable after five weeks (see the complete results and Supplementary Figs. S1 and S2 in the Supplementary Information).

#### Accuracy and reaction time results during the training

These behavioral data have been reported in our previous study^40^, but we are going to describe them briefly to emphasize the progress of the trained group on *n*-back during the training sessions. The results of the test part of the training were used, with the correct responses and RTs, and a repeated-measures 2 × 4 ANOVA was performed. Regarding accuracy, the ANOVA showed significant effects for the Session of training (*F*_(3,22)_ = 4.85 p-value < 0.05), the *n*-back level or load (*F*_(1,24)_ = 8.06 p-value < 0.05), and the Load Level × Training Session interaction (*F*_(3,22)_ = 4.07 p < 0.05). As expected, the performance on the *n*-back task improved significantly and progressively after each training session on both the 2-back and 3-back tasks. In the case of the RT values, results revealed a statistically significant effect for the Session of training (*F*_(3,22)_ = 8.62 p-value < 0.001), indicating that participants’ RTs declined significantly as the training sessions progressed. The overall results indicate that trained participants steadily improved their accuracy as they accumulated 200 minutes of training.

### Task fMRI results

#### Task effects at pre-training session

**Figure 2:**
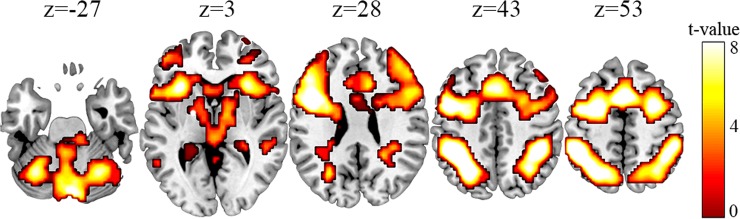
PASAT general task activations in the pre-training session. Contrast: add > repeat (active condition > control condition). A corrected FDR threshold of p-value < 0.05 (cluster correction, criterion: 52 voxels of extension) and an uncorrected threshold of p-value < 0.001 (voxel level) were employed.

In order to investigate the cerebral regions that support the performance on the PASAT task, a whole-brain, one-sample *t* test (active condition > control condition) was used; for this purpose, the data collected in the pre-training session (S1) were used. Activations were found in cortical and subcortical areas involved in working memory processes. Specially, the results reported activations in the inferior and middle parts of frontal cortical areas (bilaterally BA 6/10/44-46), which also included the anterior part of the insula (BA 13) and the inferior (BA 40) and superior (BA 7) cortical parietal regions (in both hemispheres), bilateral inferior temporal cortex (BA 20), and bilateral cerebellum. The activated midbrain areas were the thalamus and the caudate (Fig. 2). Both groups’ data were used in the analysis, which was conducted with a corrected FDR threshold of p-value < 0.05 (cluster correction, criterion: 52 voxels of extension) and an uncorrected threshold of p-value < 0.001 (voxel level).

In addition, a two-sample *t* test analysis was performed using the data collected in S1 for the purpose of verifying that the two groups had no differences in brain responses during the PASAT performance. The results showed no significant functional differences using a corrected FDR threshold of p-value < 0.05 (cluster correction) and an uncorrected threshold of p-value < 0.001 (voxel level).

#### Neural Transfer effects: Group × Session interaction analysis results

**Figure 3:**
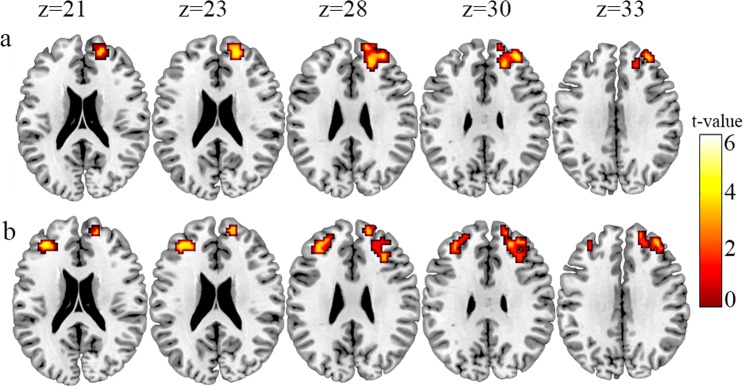
Interaction analysis results. Decreased activation was found in the prefrontal lobe during the performance of the PASAT task in the trained group after the *n*-back training. **(a)** Trained group (pre-training > post-training) > Control group (pre-training > post-training) and **(b)** Trained group (pre-training > follow-up) > Control group (pre-training > follow-up). A corrected FDR threshold of p-value < 0.05 (cluster correction, criterion: 113 voxels of extension for contrast 1 and 99 voxels of extension for contrast 2) and an uncorrected threshold of p-value < 0.001 (voxel level) were used.

To investigate neural transfer effects, we carried out three different 2 × 2 ANOVAs using Session (S2 vs S1, S3 vs S1, and S3 vs S2) as within-subjects factor and Group (Trained vs. Control) as between-subjects factor. The aim of these analyses was to study the training effects on the training participants and across sessions, while controlling for repetition effects by using between-subject controls. These analyses assessed the brain changes from pre-training to post-training and follow-up session, in terms of increases or decreases in cerebral activation (Fig. 3 and Table 1).

**Table 1:** Interaction analysis results: (**A)** comparing pre-training to post-training session and (**B**) comparing pre-training to follow-up session.

	BA	Cluster extent	MNI Space			Z-value	T-value
			x	y	z		
**(A) Trained Group (pre-training > post-training) > Control Group (pre-training > post-training)**							
R DLPFC	46	113	33	47	29	4.27	4.72
**(B) Trained Group (pre-training > follow-up) > Control Group (pre-training > follow-up)**							
L DLPFC	46	125	−33	41	20	4.14	4.54
R DLPFC	9	99	15	56	26	3.77	4.08

Corrected FDR threshold of p-value < 0.05 (cluster correction, criterion: 113 voxels of extension for contrast 1 and 99 voxels of extension for contrast 2) and an uncorrected threshold of p-value < 0.001 (voxel level). L = Left. R = Right. BA: Brodmann Area. DLPFC: dorsolateral prefrontal cortex.

The first analysis comparing S2 vs S1 was performed to investigate the immediate cerebral changes after 200 minutes of training. A corrected FDR threshold of p-value < 0.05 cluster correction, criterion: 113 voxels of extension and an uncorrected threshold of p-value < 0.001 (voxel level) were employed. When studying the effects of the trained group [contrasts: *Trained group (S1* > *S2)* > *Control group (S1* > *S2)*], the results showed that the trained participants had less activation in the right DLPFC (BA 46) than the control participants during PASAT performance in S2 (MNI coordinates were x = 33, y = 47, and z = 29, T = 4.72). No differences were found when the opposite contrast was conducted; in other words, no increased activations were found across sessions when the participants who completed the training were compared to the participants in the control group.

In order to assess the brain changes from pre-training to follow-up session, a second ANOVA comparing S3 vs S1 was performed. A corrected FDR threshold of p-value < 0.05 cluster correction, criterion: 99 voxels of extension and an uncorrected threshold of p-value < 0.001 (voxel level) were used. When studying the effects of the trained group [contrasts: *Trained group (S1* > *S3)* > *Control group (S1* > *S3*)], the results showed a significant difference in the left DLPFC (BA 46; x = −33, y = 41, and z = 20, T = 4.54) and right DLPFC (BA9; x = 15, y = 56, and z = 26, T = 4.08), indicating less activation in this area in the trained group compared to the control participants during S3. Again, the opposite contrast did not yield any statistically significant differences.

Finally, a third ANOVA comparing S3 vs S2 was performed to evaluate the maintenance of immediate changes from the *n*-back training as time passed. No significant effects were found in either condition or any possible direction (contrast) within the comparison; that is, the decreased activation found from S1 to S2 remained stable in S3 without additional changes.

#### PASAT and *n*-back activation spatial overlap

**Figure 4:**
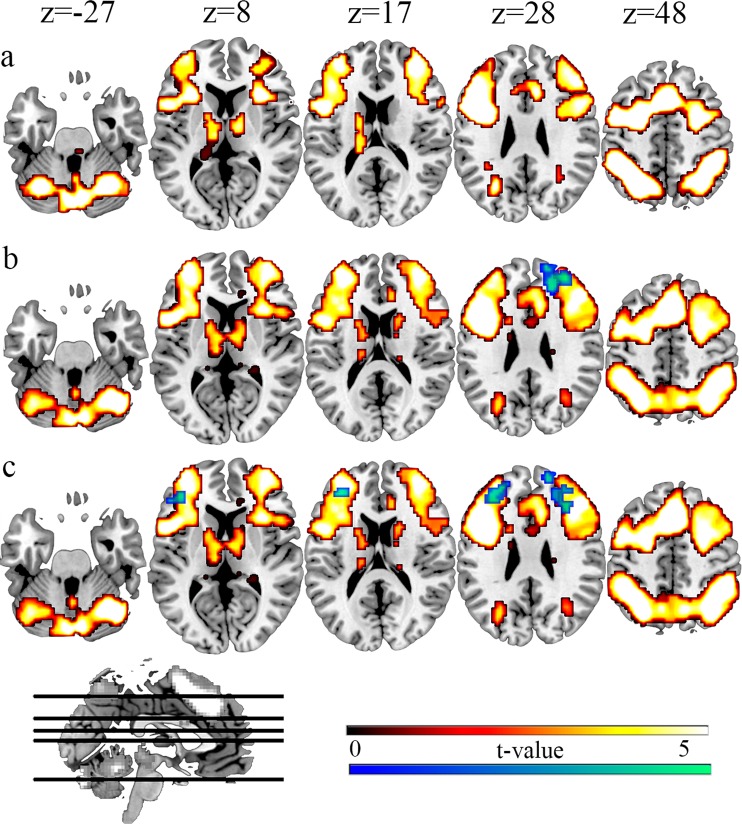
Regions in which overlapping activation were observed between the training (*n*-back) and the transfer task (PASAT). (**a**) Conjunction analysis between *n*-back (contrast: *2-back* + *3back* > *0-back)* and PASAT (contrast: *add* > *repeat)*. The shared areas activated during the performance of both tasks are colored in red. A corrected FDR threshold of p-value < 0.05 (cluster correction, criterion: 45 voxels of extension) and an uncorrected threshold of p-value < 0.001 (voxel level) were used. (**b**) Overlap of the *n*-back general task activation in the trained group in S1 with the decreased activation found in the DLPFC during PASAT performance in S2 (the same as the Fig. 3a). The yellow scale represents the contrast: *2-back* + *3back* > *0-back*, and the blue scale represents the contrast: Trained group (pre-training > post-training) > Control group (pre-training > post-training). A corrected FDR threshold of p-value < 0.05 (cluster correction, criterion: 1500 voxels of extension for *n*-back and 113 voxels of extension for PASAT) and an uncorrected threshold of p < 0.005 (voxel level) for *n*-back and a p-value < 0.001 (voxel level) for PASAT were utilized. (**c**) Overlap of the *n*-back general task activation in the trained group in S1 with the decreased activation found in the DLPFC during PASAT performance in S3 (the same as the Fig. 3b). Therefore, the yellow scale represents the contrast: *2-back* + *3back* > *0-back*, and the blue scale represents the contrast: Trained group (pre-training > follow-up) > Control group (pre-training > follow-up). We employed a corrected FDR threshold of p-value < 0.05 (cluster correction, criterion: 1500 voxels of extension for *n*-back and 99 voxels of extension for PASAT) and an uncorrected threshold of p < 0.005 (voxel level) for *n*-back and a p-value < 0.001 (voxel level) for PASAT).

To verify that PASAT and *n*-back involve the same brain areas, a conjunction analysis of the two tasks was conducted using both groups’ data collected in S1. A corrected FDR threshold of p-value < 0.05 (cluster correction, criterion: 45 voxels of extension) and an uncorrected threshold of p-value < 0.001 (voxel level) were employed. Figure 4a shows the common brain areas activated during the performance of both tasks: inferior, middle, and superior cortical frontal regions in both hemispheres (BA 6, 8-11, 32, 45–48), the supplementary motor area (SMA) and the anterior part of the cingulum (ACC) (BA 6 and BA 32) and the anterior part of the insular cortex (BA 13), inferior and superior lateral and medial cortical parietal regions (BA 7 and BA 40, bilaterally), bilateral inferior temporal cortex (BA 20), bilateral crus I of the cerebellum, and two subcortical areas (globus pallidus and thalamic nuclei). Table 2 describes the global maximum brain activations of the conjunction analysis. Additionally, in order to ascertain that our results showing decreased activation during the performance of the PASAT matched the activations on the *n*-back trained task, an overlap between the results of the *n*-back general task activation in the trained group and the interaction analyses (stated in previous paragraphs) in S2 (Fig. 4b) and in S3 (Fig. 4c) was also included. The *2-back* + *3-back* > *0-back* contrast was used to procure the task-activation map for the spatial overlap. Finally, the brain maps were saved and overlaid in MRICron over the template ch2bet.nii.

**Table 2:** List of global maximum brain activations as a result of the conjunction analysis between *n*-back (contrast: 2-back + 3back > 0-back) and PASAT (contrast: add > repeat).

	BA	Cluster extent	MNI SPACE			Z-value	T-value
			x	y	z		
L Parietal Inferior	40	2104	−42	−43	44	Inf	12.99
L Precentral	6	5079	−45	5	35	Inf	10.51
R Cerebellum		847	33	−58	−31	7.44	8.61
		230	3	−25	−10	5.08	5.43
L Thalamus		187	−12	−16	11	4.91	5.23
L Occipital Inferior	37	56	−48	−58	−13	4.87	5.18
R Temporal Inferior	37	45	60	−49	−10	4.48	4.73

Corrected FDR threshold of p-value < 0.05 (cluster correction, criterion: 45 voxels of extension) and an uncorrected threshold of p-value < 0.001 (voxel level). L = Left. R = Right. BA: Brodmann Area.

## Discussion

In the current study, the behavioral and neural far transfer effects from a working memory training task (*n*-back) to another untrained working memory task (PASAT) were examined. Previous literature has found mixed evidence supporting transfer effects, and whether or not the tasks share the same brain systems seems to be a determinant in observing these effects. In the present study, we tested whether two robust but clearly different working memory tasks support the far transfer effect phenomenon. Although the behavioral analysis results show no global transfer effects, the brain imaging analysis results indicate that neural transfer effects have occurred. These effects consisted of a decrease in activation in the anterior DLPFC, and they were found immediately after training and five weeks later. These results demonstrate that a working memory training program improves the neural processing of an untrained task that shares the use of the central executive, suggesting some kind of improved neural efficiency.

Our results showed that training was effective in improving performance on the trained task, but not on the transfer task. The use of an easy version of the PASAT task (i.e. 3 second inter-stimulus interval) allowed us to minimize the possible bias of performance differences on brain activations; that is, we obtained a homogenous performance across subjects during baseline and post-training scans. The mean accuracy at baseline was high, but it allowed significant and small improvements (i.e. 3%) in post-training sessions. This mean improvement was equal for both groups and may be mainly due to the retest effect because repeated exposure to the PASAT has previously demonstrated significant increases in performance^36^. This means that only some of the participants in the trained group behaviorally showed a significant transfer effect from *n*-back training, whereas the performance of the others did not improve due to training or retest. In this regard, our experiment adds to others that have failed to observe behavioral far transfer between working memory tasks after training^12,16–18,41^.

In relation to brain activity, PASAT task activations mainly include frontal and parietal areas related to working memory. This pattern coincides with previous neuroimaging studies that used the PASAT^34,38,39^, and it strongly overlaps with the *n*-back task activation pattern, as shown in Fig. 4a. The overlap with the *n*-back affects the bilateral DLPFC, SMA/ACC, insula, bilateral parietal cortex, bilateral inferior temporal cortex, bilateral cerebellum, and two midbrain areas (thalamus and globus pallidus). Both the PASAT and *n*-back are working memory tasks where participants have to maintain verbal information for a short period of time and give a response^35,36^. However, the tasks differ substantially in the type of stimuli, the kind of cognitive manipulations (on the PASAT they have to add simple numbers, and on the *n*-back they have to retain a different number of letters), and the type of response (verbal or manual), preventing similar processes controlled by posterior areas of the brain. Thus, the general neural overlap represents the common use of the working memory network, but the cognitive processes involved in each task only have in common the need to maintain verbal information, a process that is more related to the central executive component of working memory. In this regard, the present results are consistent with previous data showing cross-modal transfer effects on working memory and other cognitive tasks (similar improvement, and using the same brain areas on the visual discrimination test^8,22^).

As far as we know, this is the first study to show the neural transfer effects located in crucial areas involved in working memory, such as the anterior prefrontal cortex. The anterior DLPFC cortex acts as the central executive system in working memory, with a flexible role that operates at the abstract level, modulating the processing of posterior brain structures^24,42^. Importantly, the area obtained overlapped with both the 2-back and 3-back and the PASAT, indicating that this area participates in both tasks. Considering the cognitive processes involved in both tasks, the maintenance of verbal information for seconds is the only process they have in common, and this process is controlled from the anterior DLPFC. Thus, we have verified the hypothesis that transfer effects occur if the training task and the transfer task involve the same brain regions and cognitive processes.

There is an intense debate about how to interpret this decrease in neural activation after training^43^. These recent views have a critical vision of the neural efficiency explanation, where participants seemed not to need so many brain resources and effort to produce less mistakes, classifying it like simply and weakly related to the underlying neurobiological mechanisms. The review by Constantinidis and Klingberg^24^ compared the effects of working memory training using neurophysiological recordings in non-human primates and neuroimaging data in humans. Whereas data on primates shows that training increases the activity of prefrontal neurons and the strength of connectivity within the prefrontal cortex, human data shows a decreased activation on the trained task after training, which was typically interpreted as indicating increased neural efficiency^27,44^. When analyzing all the literature, in primate studies increases were found in the ventrolateral, but not the DLPFC^24^, whereas results in humans were found in dorsolateral areas. Therefore, given the lack of transfer behavioral effects, what does the DLPFC activation decrease reflect? This result may be consistent with the neural efficiency interpretation because, although there were no significant behavioral differences between groups on the PASAT, trained participants improved their performance after training on both tasks. Because the DLPFC is involved in top-down control^26^, the decrease may reflect a lower cognitive control requirement after training to attain a correct response. Thus, we can tentatively interpret the present results as increased neural efficiency. Importantly, the decreases observed after training persisted with no change after five weeks without training. Previous research found stability effects on the trained task (*n*-back;^29,40^), but this is the first time the stability of neural transfer effects has been demonstrated.

On the other hand, and taking into account other neural transfer studies, our results differ significantly from studies reporting neural near transfer in the striatum^31,32^. These studies used two similar tasks requiring the person to learn certain cognitive processes, such as letter updating^31^ or dual-task working memory^32^, which are transferred from the trained to the transfer task. The striatum is involved in these specific transfers that require the regulation of the information that is relevant to the working memory task. This process is not involved in the far transfer in our experiment. In fact, unless we used an easy version of the task, the PASAT is a demanding task with a clear component of cognitive control (involving maintenance of stimuli and, probably, inhibition of responses), and this is the process that is probably transferred from the *n*-back training.

Limitations of the current study involve the control group and the consecutive presentation of two different task in the fMRI sessions. On the one hand, the use of a control group that did not complete any task between pre-training and post-training may cause differences in motivation between the participants with regard to their efficiency in completing the cognitive tasks. The gains found in the trained group might not be produced by working memory or the *n*-back training itself, but rather by receiving training in general. Some authors have stated that having a proper active control condition is necessary when testing hypotheses about transfer^21^. However, Au *et al*.^19,20^ and Soveri *et al*.^7^, in their meta-analysis, did not find significantly different training effects depending on the type of control group. Moreover, the fact that neural changes remain after five weeks of no training increases the possibility of a general effect of training. Nonetheless, future investigations should use control groups that complete a different task in their experimental designs. On the other hand, the use of *n*-back immediately prior to the PASAT task may influence subsequent activation. Nevertheless, the lack of feedback during the *n*-back task and the lack of variability in the behavioral measures minimize the possible impact of *n*-back on PASAT. Moreover, the experimental design utilized here is similar to procedures used in previous behavioral and fMRI studies designed to investigate both direct and transfer effects^30–32^ and, to our knowledge, no specific study has shown the possible influence of one task on another.

In conclusion, our results showed far transfer neural effects on the PASAT task. *N*-back training caused a decrease in the activation of the DLPFC during PASAT performance, which indicates a clear neural transfer effect. These cerebral changes remained stable after five weeks. These results point in the direction of the hypothesis that transfer will occur if the training and the transfer task involve the same brain regions and processing components. Future research should focus on how this kind of working memory regime affects a clinical population, and test whether the stability of these effects lasts longer than five weeks.

## Materials and Methods

The data sample and part of the methodology used in the present work is the same that we used in Miró-Padilla *et al*.^40^. The former study was focused on studying behavioral and task-fMRI brain changes from the trained task (*n-*back). For this reason, details regarding the *n*-back fMRI and training task design, behavioral analysis, and results are reported in our previous study^40^ and can be found in the Supplementary Information.

### Participants

Fifty-two healthy right-handed undergraduates (20 male) recruited from the population of students at the Universitat Jaume I participated in this study. None of these students indicated previous neurologic or psychiatric illness. The investigation was approved by the Ethical Committee of the Universitat Jaume I, and it was performed in full accordance with their relevant guidelines and regulations. Each subject gave his/her written informed consent prior to scanning. Their active participation was rewarded with monetary compensation. Subjects were randomly allocated to an experimental group (trained group) (N = 25, mean age = 22.72 ± 1.51, 10 men) or to a control group (N = 27, mean age = 22.52 ± 1.45, 10 men). Intellectual level was evaluated with the Matrix Reasoning Test (Wechsler Adult Intelligence Scale version III-R), the mean direct score on the test was 21.08 (standard deviation: 3.35), and for the control group, the mean direct score was 21.63 (standard deviation: 1.94). Between-group differences in gender distribution, age, and IQ were non-significant. The only difference between the two groups was the training (the control group did not do anything).

### Experimental design

Using an adapted block-design *n*-back task^40^ followed by a block-design PASAT task, both groups participated in three fMRI sessions with identical procedures. The second fMRI session took place seven days after the first one, and the last session took place 35 days (five weeks) after the second session. The trained group trained 200 minutes between the two first scanner sessions on an *n*-back task, and we used the adaptive method. Although the training time could be a factor influencing the training results, the length of the training protocol used in the present study is similar in training time to previous studies^8,35,45–47^. In relation to this factor, some authors provide support for the usefulness of short periods of single *n*-back task training. Finally, a brief single *n*-back training has chosen with an eye on future clinical procedures, considering that implementing a long training protocol might be demanding and expensive for patients and organizations. For this reason, test the effects of this variety of short working memory training protocol on healthy controls was our objective in order to allow comparisons with clinical populations in future investigations. Neither group had training between the two last sessions. The pre-training session, post-training session, and follow-up session are considered Session one (S1), Session two (S2), and Session three (S3), respectively. Auditory stimuli (numbers) were presented with the professional version 2.0 of E-Prime developed by Psychology Software Tools (Pittsburgh, PA) at a HP laptop workstation (with a screen resolution of 800 × 600 and a 60 Hz refresh rate). Subjects looked at the laptop screen, listened to the stimuli, and provided their responses with goggles, headphones, and response-grips compatible with MRI (these devices are VisuaStim, developed by Resonance Technology, Northridge, CA). In addition, a microphone compatible with MRI was used (the device is FORMIII developed by Optoacoustics, Inc.). Therefore, we collected the participants’ oral and manual responses. Sound volume was adjusted so that each participant could hear the stimuli properly, and scanner noise was cancelled. The RTs and accuracy scores were saved throughout the tasks.

#### PASAT fMRI task

Participants completed six-minute versions of the auditory PASAT task, which included six one-minute blocks. Three of these six PASAT blocks belonged to the control condition (repeat), and three belonged to the activation task (add). The subjects heard a sequence of numbers, ranging from one to nine, at a rate of one number every three seconds (19 stimuli per block). During the control task, participants were instructed to repeat each number in a presented series aloud. The activation task consisted of adding the first number to the second, the second to the third, and so on. They calculated the sum of the last two numbers and responded aloud^48^. We collected the number of correct responses; 54 was the maximum accuracy score per task (18 per block). Subjects were told to answer as quickly as possible while avoiding making mistakes, and they were given oral instructions about how to perform the task. To familiarize themselves with the stimuli presentation and how to respond, they performed a four-minute practice task outside the scanner that was composed of four blocks, two per condition. A similar laptop with the same display configuration and hardware as the one used to present the in-scanner task was used for the oral responses.

### Neuroimaging data acquisition and preprocessing

A 1.5 T scanner by Siemens Symphony (Erlangen, Germany) was used to collect functional MRI data. The sequences were the same during the pre-, post- and follow-up sessions. Three different sequences were acquired in each session: first, a high-resolution structural T1-weighted MPRAGE; then, the images performing the *n*-back task; and finally, the PASAT performance. Subjects took a supine position in the scanner. Fixation padding was used to reduce motion degradation. Furthermore, participants were asked to minimize head movement, even while giving the answers. The functional images were acquired using a gradient-echo with a T2*-weighted echo-planar sequence that covered the entire brain (TR/TE = 2500/49 ms, matrix = 64 × 64 × 28, flip angle = 90°, voxel size = 3.5 × 3.5 × 4.48; slice thickness = 4 mm; slice gap = 0.48 mm) on the task-fMRI to obtain 270 volumes for *n-*back and 146 volumes for PASAT. All the scanner acquisitions were made in parallel to the anterior–posterior commissure plane. Prior to the fMRI sequences, a high-resolution structural T1-weighted MPRAGE sequence was obtained (TR = 2200 ms; TE = 3 ms; flip angle 90°, matrix = 256 × 256 × 160; voxel size = 1 × 1 × 1 mm).

SPM12 (developed by Wellcome Trust Centre for Neuroimaging in London, UK) was used for preprocessing and to perform the statistical analyses of the fMRI data. Each subject’s fMRI data were aligned to the AC-PC plane by employing his/her anatomical image. To fit the mean functional image, each functional image would be realigned and resliced based on the head motion correction. None of the participants had a head motion greater than 2.5 mm maximum displacement in any direction or 2.5° of any angular motion during the entire scan. Next, the mean functional image was co-registered with the anatomical image (T1-weighted), then re-segmenting the adapted anatomical image. During the normalization to the MNI (Montreal Neurological Institute, Montreal, Canada), the functional images were spaced at a 3 mm^3^ resolution, and they were spatially smoothed using an isotropic Gaussian kernel of 8 mm full-width at half-maximum (FWHM).

### Accuracy analysis

IBM SPSS Statistics software (Version 22 Armonk, New York, USA) was utilized in order to process the behavioral data (accuracy of participants’ performance). An analysis of variance (ANOVA) with Session as within-subject factor (3 levels: S1, S2 and S3) and Group as between-subject factor (2 levels: Trained group and Control group) was performed. With the data from the testing section of the training, a repeated-measures 2 × 4 ANOVA was performed, and Load Level (2-back vs. 3-back) and Training Session (1 vs. 2 vs. 3 vs. 4) were used as within-subject factors. For detailed information about the *n-back training task* and *n-back fMRI behavioral analysis* please see the data presented in the Supplementary Information provided.

### Neuroimaging analysis

#### fMRI-task postprocessing analysis: first level of analysis

Employing SPM12, a General Linear Model was used to carry out the statistical analyses^49^ for each participant and time point,. In the first-level analysis, the conditions of interest corresponding to *add* > *repeat* (active condition > control condition) were modeled. To estimate the BOLD signal, the stimuli onset and the canonical hemodynamic response function were convolved. Six motion realignment parameters were used to explain signal variations produced by head motion, in other words, as covariates of no interest. In order to eliminate the low-frequency components, a high-pass filter (128 s) was applied to the functional data. Then, we used the contrast images obtained to directly compare the conditions of interest. In the cross-sectional analysis, we compared the S1 conditions of interest with the purpose of evaluating differences on the PASAT task before learning.

#### fMRI-task postprocessing analysis: Group × Session interaction analysis

Due to the use of multiple comparisons in the different fMRI analyses, in all of the fMRI analyses performed in the present work a FDR approach was used to control the proportion of false positive voxels (type I errors) among those voxels that were considered positive results, using the SPM12 software (Wellcome Trust Centre for Neuroimaging in London, UK; see also:^50,51^). Specifically, we used a corrected FDR threshold of p-value < 0.05 (the specific cluster correction is indicated in the results) and an uncorrected threshold of p-value < 0.001 (voxel level).

A whole-brain one-sample *t* test was performed in the cross-sectional analysis in order to investigate the regions of the brain involved in the PASAT task *(active condition* > *control condition)*, employing the fMRI data gathered in S1. Additionally, to assess the equality of the brain responses in the two groups, images collected in S1 were utilized to perform a two-sample *t* test. Thus, any between-group brain differences observed in the following sessions would be produced by training effects. A longitudinal analysis was carried out in the second-level analysis in order to evaluate: the immediate effect of training, comparing post-training and pre-training sessions; the long-term effects of training, comparing follow-up and pre-training sessions; and the differences between immediate and long-term effects, comparing follow-up and post-training sessions. For this purpose, an interaction analysis was conducted between sessions by means of three separate 2 × 2 ANOVAs, with session (S2 vs S1, S3 vs S1 and S3 vs S2) as within-subjects factor and group (Trained vs. Control) as between-subjects factor.

#### fMRI-task postprocessing analysis: conjunction analysis

To identify the brain regions that were commonly activated in the pre-training session by the training task (*n*-back) and the transfer task (PASAT), a conjunction analysis (conjunction null) was performed. This analysis is a method based on the minimum statistic general linear model used to determine whether two tasks activate the same regions of the brain. This analysis retains voxels with main effects of condition, but a “null” interaction effect between them, in other words, an absence of differences between conditions^52–54^. Both groups’ S1 data were utilized to perform this analysis. The conjunction analysis included, for the training task, the contrast [*2-back + 3back > 0-back*], and for the transfer task, the contrast [*add* > *repeat*]. The analysis was thresholded using a corrected FDR of p-value < 0.05 (cluster correction, criterion: 45 voxels of extension) and an uncorrected threshold of p-value < 0.001 (voxel level).

### Ethical approval

The study was approved by the institutional Ethics Committee of the Universitat Jaume I.

### Informed consent

All the participants provided written informed consent prior participation.

## Supplementary information

Supplementary Information.
