# Supplementary material for: Locating neural transfer effects of n-back training on the central executive: a longitudinal fMRI study
Source: Sci Rep. 2020 Mar 23;10:5226. doi: 10.1038/s41598-020-62067-y (PMC7089996; doi:10.1038/s41598-020-62067-y)

SUPPLEMENTARY INFORMATION

Title: Locating neural transfer effects of *n*-back training on the central executive: a longitudinal fMRI study

Authors: Anna Miró-Padilla (amiro@uji.es), Elisenda Bueichekú (bueichek@uji.es), César Ávila (avila@uji.es).

The data sample used in the present work is the same data sample used in Miró-Padilla et al. (40). The former study was focused on studying behavioral and task-fMRI brain changes from the trained task (*n*-back). For this reason, details regarding the *n*-back fMRI and training task design, behavioral analysis, and results presented here were firstly reported in our previous study (40).

METHODS

N-Back fMRI task

Three load levels composed the task: 0-back (the baseline control task), 2-back and 3-back which were the working memory blocks. Participants responded "yes" on the 0-back when the letter X (the target) appeared on the screen and "no" when there were no targets. On the working memory blocks, when the current letter shown on the screen matched the one presented 2 or 3 items back subjects responded "yes"; and "no" to any other letters. Using their right hand, participants gave manual responses responding to targets with their thumb and to non-targets with their forefinger.

The task lasted 11 minutes and was composed by nine blocks, three for each load level and did not contain any lures. Each block lasted 60.7 seconds, consisting in: 200 ms of a blank screen, 30 (6 target) consecutive trials of single letter stimuli (500 ms duration, 1500 ms inter-stimulus interval) and 500 ms of a blank screen at the end. Before each block, we added a fixation cross (8000 ms) and an instruction display (2000 ms) indicating task difficulty (0-back, 2-back or 3-back). In total, 270 stimuli (54 of them targets) pseudo-randomized composed the task. The stimuli were 15 different capital letters from the alphabet (B, C, D, F, G, H, J, L, N, P, Q, R, S, T and V). On 2- and 3-back any letter could be a target, but only the "X" letter was the target on 0-back.

Participants watched the letters, instructions, and fixation point (all black ink with a 54-point Arial font)in the middle of the screen on a white background.

To familiarize themselves with the stimuli presentation and how to respond, they performed a five-minute practice task outside the scanner that was composed of three blocks, one per load level. A similar laptop with the same display configuration and the hardware as the one used to present in-scanner task was used for the oral responses. Subjects were told to answer as quickly as possible, but avoiding making mistakes, and they were given oral instructions about how to perform the task.

N-back training task

Four consecutive training sessions (TS) of single *n*-back were conducted by the trained group after fMRI in S1. They came to our laboratory located at the University and carried out only one TS per day. Each TS were organized in two phases and lasted 60 minutes: the learning part and the testing part. In the learning part that lasted 50 minutes, subjects carried out an adaptive *n*-back paradigm adapted from (Jaeggi, Buschkuehl, Jonides & Perrig, 2008), whereas in the testing part, they performed a simple *n*-back task that lasted 10 minutes. Hence, the total training time was approximately 200 minutes, plus 40 minutes for the testing part. Their results on the test part were useful to evaluate their progress on the task. The same laptop as in the fMRI sessions was used, and their responses were collected via response-grips (NordicNeuroLab, Bergen, Norway).

The adaptive *n*-back task was composed by three active load levels (1-, 2- and 3-back) using the same stimuli and block timing as in the *n*-back fMRI task and lasted approximately 16 minutes. Participants carried out three runs per TS. Feedback were given to the subjects after each stimulus and at the end of each block about their accuracy and time reaction. For motivational reasons, we changed the level of difficulty by changing the level of “n” (1, 2, or 3). After each block, the participant’s individual performance was analyzed, and the *n*-back level was automatically adjusted up to a maximum of 3-back. Thus, if the participant had at least 90% correct answers, the level of “n” in the next block was increased by one, but it was decreased by one if accuracy was below 80%. The *n*-level remained constant in all other cases. In the last run, we increased the percentage by five percent to make it more difficult. Therefore, if the participant had at least 95% correct answers, the level of “n” was increased by one,

whereas it was decreased by one if accuracy was below 85%. Each run started with the minimum level of “n”. For the feedback: a colored circle appeared for a few seconds at the corner of the screen after each response: green if the answer was correct, red if it was a mistake, and blue in omissions. Also, correct response percentage and reaction time average was given to participants at the end of each block. On the test part which lasted 10 minutes and without feedback, subjects carried out an eight-block *n*-back task, half of 2-back and half of the 3-back. To each stimulus, the E-Prime software collected each participant’s accuracy and reaction time (RT).

N-back fMRI behavioral analysis

In order to process the behavioral data (accuracy and RTs for participants’ performance) IBM SPSS Statistics software (Version 22 Armonk, New York, USA) was used. For each variable, a repeated-measures 2x3x3 mixed model ANOVA was conducted. Group (training x control) as the between-subjects factor and Load Level (0-back vs. 2-back vs. 3-back) and Session (1 vs. 2 vs. 3) as within-subjects factors were used. Also, post-hoc analysis was performed for each variable. A repeated measures 2x4 ANOVA with the test part data of the training was conducted, with Load Level (2-back vs. 3-back) and Training Session (1 vs. 2 vs. 3 vs. 4) as within-subjects factors.

RESULTS

These data have been reported in our previous study (40).

N-back behavioral fMRI results

The repeated measures 2x3x3 mixed-model ANOVA conducted for accuracy yielded main effects for Session ($F_{(2,50)} = 34.66$ $p<.001$) and Load Level ($F_{(2,50)} = 42.85$ $p<.001$), which means that all the participants reduced their mistakes in the post-training and follow-up sessions, compared to Session 1, and that the highest accuracy scores were observed during the 0-back. These main effects were driven by significant Group x Session ($F_{(2,50)} = 7.77$ $p=.001$), Load Level x Session ($F_{(4,48)} = 13.07$ $p<.001$), and Load Level x Group ($F_{(2,50)} = 7.23$ $p=.002$) interactions. The first interaction indicated that trained participants were better than controls during the post-training and follow-up sessions. The second indicated that differences between load levels were greater at pre-training, whereas the third reflected that the training group showed better performance

than the control group on 2-back and 3-back. As expected, the Load Level x Session x Group interaction reached significance ($F_{(4,48)}$ = 4.01 p=.007), which means that the trained group became more accurate in the post-training and follow-up sessions than the control group, when performing the 2-back and 3-back load levels (see **Supplementary Fig. S1**). Post-hoc analyses revealed that these differences were significant for 3-back vs 0-back (p=.002), and they only approached significance (p=.13) for 2-back vs 0-back (40).

Analyses of RTs scores revealed a similar pattern to the one found for accuracy. The 2x3x3 ANOVA also yielded significant main effects for Session ($F_{(2,50)}$ = 51.59 p<.001) and Load Level ($F_{(2,50)}$ = 75.37 p<.001). Both groups responded faster in the post-training and follow-up sessions than in the pre-training session. Moreover, both responded faster in the 0-back load level compared to the 2-back load level, as well as in the 2-back load level compared to the 3-back load level. Significant two-way interactions were obtained for the Group x Session ($F_{(2,50)}$ = 28.14 p<.001) and Load Level x Session ($F_{(4,48)}$ = 28.23 p<.001) interactions. The first two interactions may be interpreted similarly to accuracy; participants were faster than controls during the post-training and follow-up, and the differences between load levels were greater at pre-training. Importantly, all these significant effects were qualified by the three-way Load Level x Session x Group interaction, which was highly significant ($F_{(4,48)}$ = 11.34 p<.001). As expected, this interaction showed that the training group, compared to the controls, was faster after training and in the follow-up session in the 2-back and 3-back load levels (see **Supplementary Fig. S2**). Post-hoc analyses revealed that this effect was significant (p<.001) for both 2-back vs 0-back and 3-back vs 0-back load levels (40).

SUPPLEMENTARY FIGURES

Supplementary Fig. S1: Results of the *n*-back accuracy behavioral analysis. Correct-response percentage per session have been plotted as a function of load level and time. Pre-training session, post-training session and follow-up session correspond to Session 1, Session 2 and Session 3, respectively. Training group data correspond to the dark gray bars and control group data to the light gray bars. Error bars represent standard error. Adapted from (40, Fig. 3).

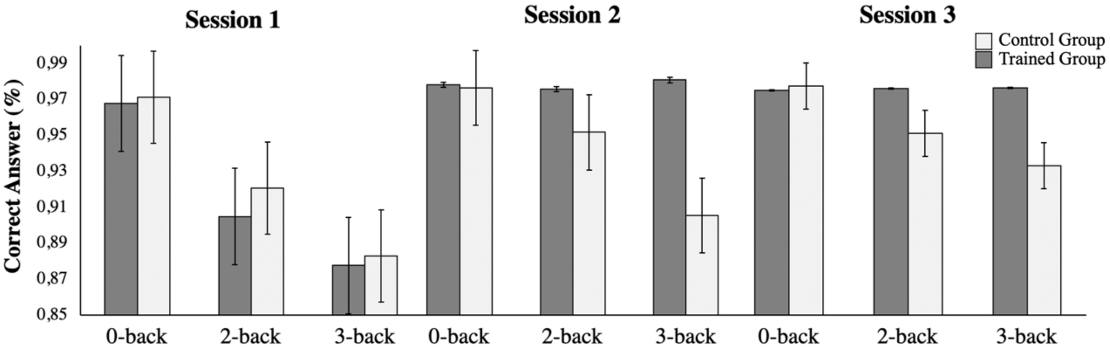

Supplementary Fig. S2: Results of the *n*-back RTs behavioral analysis. Mean reaction times (in milliseconds) per session have been plotted as a function of load level and time. Pre-training session, post-training session and follow-up session correspond to Session 1, Session 2 and Session 3, respectively. Training group data correspond to the dark gray bars and control group data to the light gray bars. RTs = Reaction Times. Error bars represent standard error. Adapted from (40, Fig. 3).

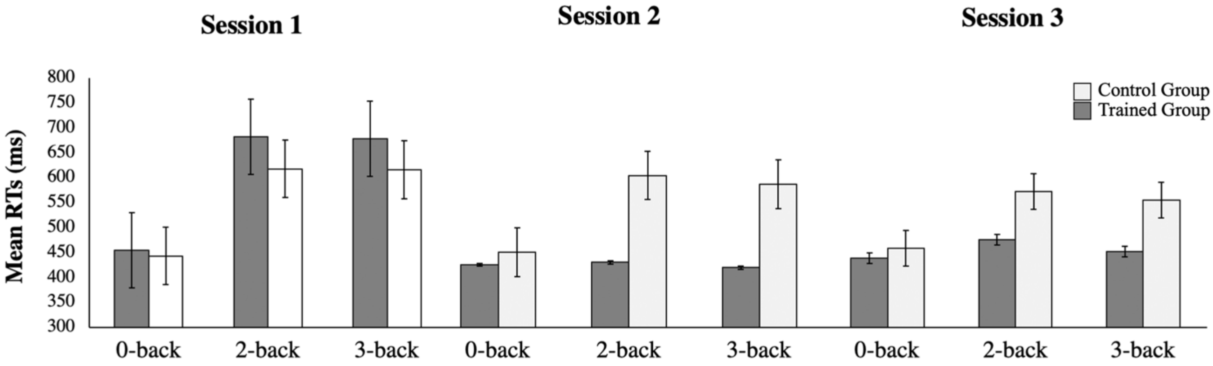

Supplement: Supplementary file 1 — Supplementary Information. [file 41598_2020_62067_MOESM1_ESM.pdf]
